# Establishment and validation of a novel prognostic model for lower-grade glioma based on senescence-related genes

**DOI:** 10.3389/fimmu.2022.1018942

**Published:** 2022-10-21

**Authors:** Junsheng Li, Jia Wang, Dongjing Liu, Chuming Tao, Jizong Zhao, Wen Wang

**Affiliations:** ^1^ Department of Neurosurgery, Beijing Tiantan Hospital, Capital Medical University, Beijing, China; ^2^ China National Clinical Research Center for Neurological Diseases, Beijing, China; ^3^ Center of Stroke, Beijing Institute for Brain Disorders, Beijing, China; ^4^ Beijing Key Laboratory of Translational Medicine for Cerebrovascular Disease, Beijing, China; ^5^ Beijing Translational Engineering Center for 3D Printer in Clinical Neuroscience, Beijing, China; ^6^ Department of Neurosurgery, The Second Affiliated Hospital of Soochow University, Suzhou, China; ^7^ Savaid Medical School, University of the Chinese Academy of Sciences, Beijing, China

**Keywords:** senescence, lower-grade glioma, signature, prognostic model, biomarker

## Abstract

**Objective:**

Increasing studies have indicated that senescence was associated with tumorigenesis and progression. Lower-grade glioma (LGG) presented a less invasive nature, however, its treatment efficacy and prognosis prediction remained challenging due to the intrinsic heterogeneity. Therefore, we established a senescence-related signature and investigated its prognostic role in LGGs.

**Methods:**

The gene expression data and clinicopathologic features were from The Cancer Genome Atlas (TCGA) database. The experimentally validated senescence genes (SnGs) from the CellAge database were obtained. Then LASSO regression has been performed to build a prognostic model. Cox regression and Kaplan-Meier survival curves were performed to investigate the prognostic value of the SnG-risk score. A nomogram model has been constructed for outcome prediction. Immunological analyses were further performed. Data from the Chinese Glioma Genome Atlas (CGGA), Repository of Molecular Brain Neoplasia Data (REMBRANDT), and GSE16011 were used for validation.

**Results:**

The 6-SnG signature has been established. The results showed SnG-risk score could be considered as an independent predictor for LGG patients (HR=2.763, 95%CI=1.660-4.599, P<0.001). The high SnG-risk score indicated a worse outcome in LGG (P<0.001). Immune analysis showed a positive correlation between the SnG-risk score and immune infiltration level, and the expression of immune checkpoints. The CGGA datasets confirmed the prognostic role of the SnG-risk score. And Kaplan-Meier analyses in the additional datasets (CGGA, REMBRANDT, and GSE16011) validated the prognostic role of the SnG-signature (P<0.001 for all).

**Conclusion:**

The SnG-related prognostic model could predict the survival of LGG accurately. This study proposed a novel indicator for predicting the prognosis of LGG and provided potential therapeutic targets.

## Introduction

Glioma was a common type of central nervous system malignant tumor (1). Based on World Health Organization (WHO) classification, the lower-grade gliomas (LGGs) referred to grade II and III gliomas, which accounted for approximately 43.2% (2, 3). Compared to glioblastomas (GBMs), LGGs were more indolent precursors with a longer median overall survival (OS) (4). However, despite the application of surgical resection, chemotherapy, and radiotherapy, as well as emerging therapies such as immunotherapy and gene therapy, the local recurrence, progression into GBM, and therapeutic quality decrease seemed to be inevitable (5, 6). And due to the different genetic backgrounds, the OS of LGG patients varied widely from 1 to 15 years (7). Although molecular markers have been applied in clinical decision-making, it was still difficult to predict the outcome of LGG precisely. Therefore, it has been necessary to investigate novel biomarkers for prognostic prediction and individualized therapeutic targets for LGG patients.

Increasing evidence indicated that cellular senescence was a key step in aging process and played an important role in tumorigenesis (8, 9). Cellular senescence was a complex stress response considered as the state of cell-cycle arrest (10–12). Cellular senescence was considered to be a protective mechanism when organisms were exposed to adverse factors, including oxidative stress, DNA damage, and oncogenic activation (13). Cellular senescence had a dual function in inhibiting and promoting tumors. On one side, cell-cycle arrest ensured tissue homeostasis and prevented tumor development with immune clearance in early tumorigenesis (14–16). Conversely, the accumulation of senescent cells promoted the senescence-associated secretory phenotype (SASP), releasing cytokines, chemokines, and growth factors and constructing a chronic inflammatory microenvironment that led to tumor progression (17–19). Furthermore, recent studies have found that cellular senescence also promoted the accumulation of various immunosuppressive cells (20). However, the predictive value and potential mechanisms of senescence-related genes (SnGs) in LGGs required further study.

We identified the dysregulated senescence-related genes in LGG and established a senescence-related predictive model with the data from TCGA database, which was further validated by CGGA databases, REMBRANDT cohort, and GSE16011 dataset. The results showed the SnG-signature was an independent risk factor for LGG survival and predicted the outcome accurately.

## Methods

### The senescence-related genes acquisition

The SnG list was obtained from the CellAge database (https://genomics.senescence.info/cells/), which contained 279 SnGs validated based on experiments (21).

### RNA-sequencing data collation and differential expression gene identification

We collected RNA-seq data from TCGA database (https://portal.gdc.cancer.gov). The 5 normal and 529 LGG samples were involved for DEG analysis. Then we used DESeq2 package to determine LGG-related DEGs (22). The inclusion criteria for DEGs has been set as |logFC|>1 and Padj<0.05. Venn analysis was performed to select overlapping genes between LGG-DEG set and SnG set. Due to the limited normal samples in TCGA, we further selected 1152 normal samples from GTEx database in UCSC XENA website (https://xenabrowser.net/datapages/) for heat mapping. Our study fully followed the publication guidelines of corresponding public databases. Then Gene Ontology (GO) and Kyoto Encyclopedia of Genes and Genomes (KEGG) analyses were performed by ClusteProfiler package to identify the main biological functions involved by DEGs (23, 24).

### Construction of SnG-related prognostic model

Univariate Cox analyses were used to find the genes with prognostic significance determined by Venn analysis. Then these genes were incorporated into LASSO regression analysis performed by glmnet package (25). The cv.glmnet function was used to select the lambda that minimized the deviations. The screened genes were eventually incorporated into the prognostic model, and the risk-score system was established. Individual LGG patients were assessed with a risk score, respectively. And the formula has been shown below (n, gene quantity; expri, gene expression value; coefficienti, gene regression coefficient).


$$\text{Risk score}=\sum_{\text{i}=1}^{\text{n}}{\text{ expr}}_{\text{i}}\times {\text{coefficient}}_{\text{i}}$$

### Prognostic model development

The mid-value of SnG-risk score divided the patients into two different risk groups. Survival package was performed to estimate the survival distributions. We included various clinical features in Cox regression and analyzed the risk score level in the subgroups. Nomogram constructed by the RMS package was used for survival prediction (26, 27).

### Gene set enrichment analysis

We used ClusteProfiler package to identify the hallmark differences based on the DEGs between the two risk groups (28). The results meeting the threshold of adjusted P-value<0.05 and FDR q-value<0.25 were considered statistically significant.

### Immunological analyses

The correlation between immune infiltration and risk score has been estimated by the single-sample Gene Set Enrichment Analysis (ssGSEA) function in GSVA package (29). A total of 24 immune cell types were included for analyses indicated previously (30). Then we evaluated the correlation between immune checkpoint expression and risk score (31).

### Validation for survival analyses

We obtained 175 LGG samples from CGGA microarray dataset, 443 LGG samples from CGGA sequencing dataset (http://www.cgga.org.cn/), 162 LGG samples from REMBRANDT cohort (http://www.betastasis.com/glioma/rembrandt/), and 107 LGG samples from GSE16011 dataset (https://www.ncbi.nlm.nih.gov/). Then the samples were divided into two different risk groups according to the criteria above, respectively. Cox regression and Kaplan-Meier analyses were used to verify the predictive value of the model.

### Statistical analyses

R project (3.6.3) was used for all our statistical analyses and graphs. Mann-Whitney U-test has been used for the risk score comparison in unpaired samples. Cox regression analyses were used for evaluate the hazard ratios (HRs) and 95% confidence intervals (CIs) evaluation. Log-rank test was used for Kaplan-Meier analyses. We defined a two-sided P value<0.05 as statistically significant.

## Results

### Identification of senescence-related genes in LGG patients and functional enrichment analyses

**Figure 1:**
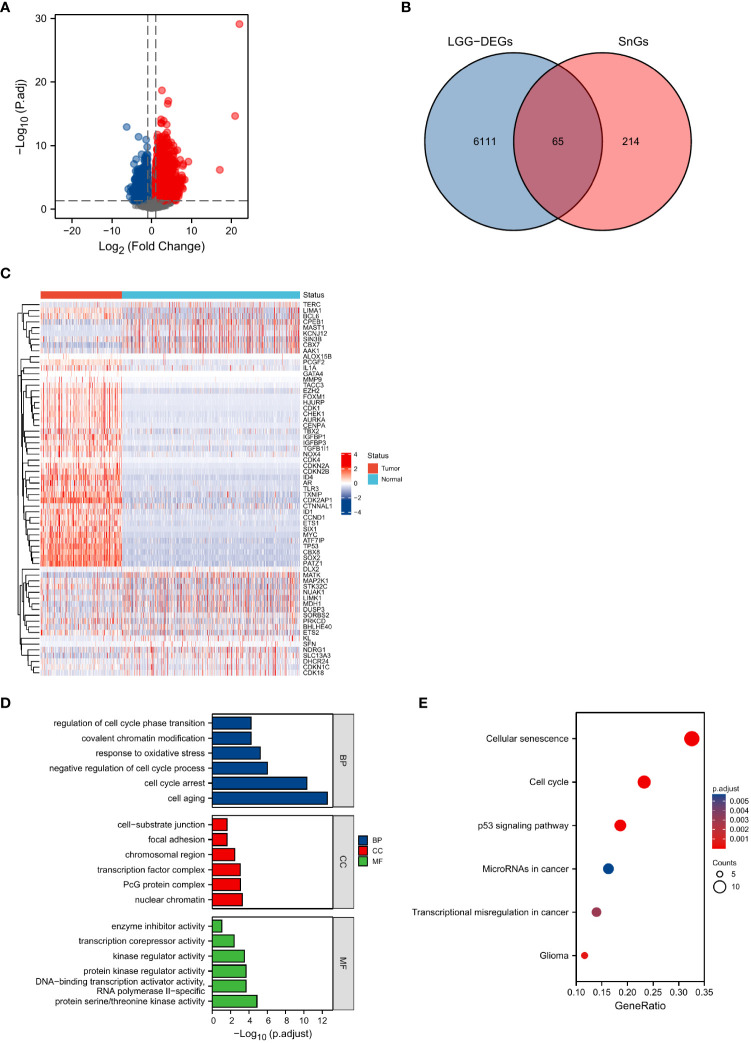
Identification and functional enrichment analyses of senescence-related DEGs. **(A)** Identification of DEGs between normal and LGG samples. **(B)** Intersections of DEGs and SnGs by Venn diagram. **(C)** Heatmap of the expression levels of the 65 senescence-related DEGs. **(D)** GO enrichment analyses. **(E)** KEGG analysis annotation.

Based on the threshold of |logFC|>1 and Padj<0.05, a total of 6176 DEGs, including 2708 upregulated genes and 3468 downregulated genes, have been identified (**Figure 1A**). We obtained 279 SnGs from the CellAge database. By overlapping the results of LGG-DEGs and SnGs, we identified a total of 65 differential expressed SnGs in LGGs for further analysis (**Figures 1B, C**).

The results of GO analyses showed that biological process (BP) included cell aging, cell cycle arrest, negative regulation of cell cycle process, response to oxidative stress, covalent chromatin modification, and regulation of cell cycle phase transition. Cellular component (CC) included nuclear chromatin, PcG protein complex, transcription factor complex, chromosomal region, focal adhesion, and cell−substrate junction. Molecular function (MF) included protein serine/threonine kinase activity, DNA-binding transcription activator activity RNA polymerase II−specific, protein kinase regulator activity, kinase regulator activity, transcription corepressor activity, and enzyme inhibitor activity (**Figure 1D**). The results of KEGG analysis included cellular senescence, cell cycle, p53 signaling pathway, microRNAs in cancer, transcriptional misregulation in cancer, and glioma (**Figure 1E**).

### Establishment of the SnG-related signature and evaluation of its prognostic role

**Figure 2:**
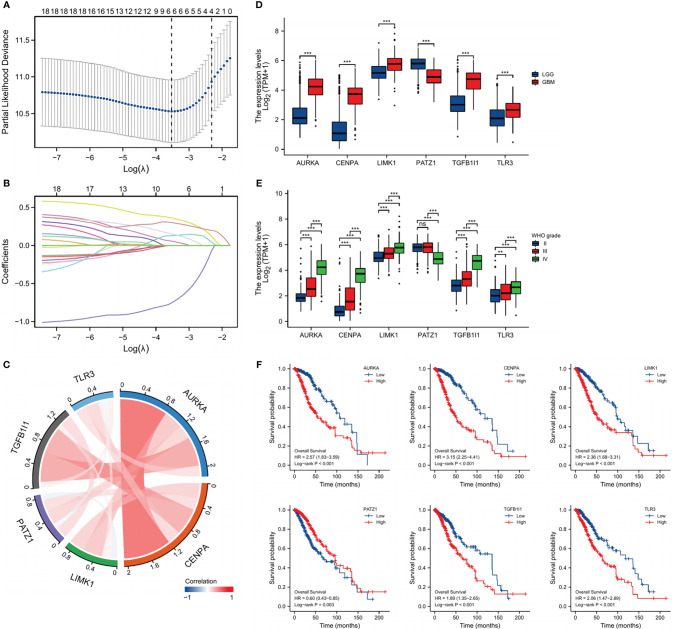
Identification of SnG-prognostic signature. **(A)** Cross-validation of the LASSO model’s parameters. **(B)** Coefficient profiles in LASSO regression model. **(C)** Correlation between the expression of the 6 SnGs. **(D)** Expression levels of the 6 SnGs in LGGs and GBMs. **(E)** Expression levels of the 6 SnGs in Grade II, III, and IV. **(F)** Survival analyses of the 6 SnGs by Kaplan-Meier curves and log-rank tests. **P<0.01; ***P<0.001; ns, not significant.

**Figure 3:**
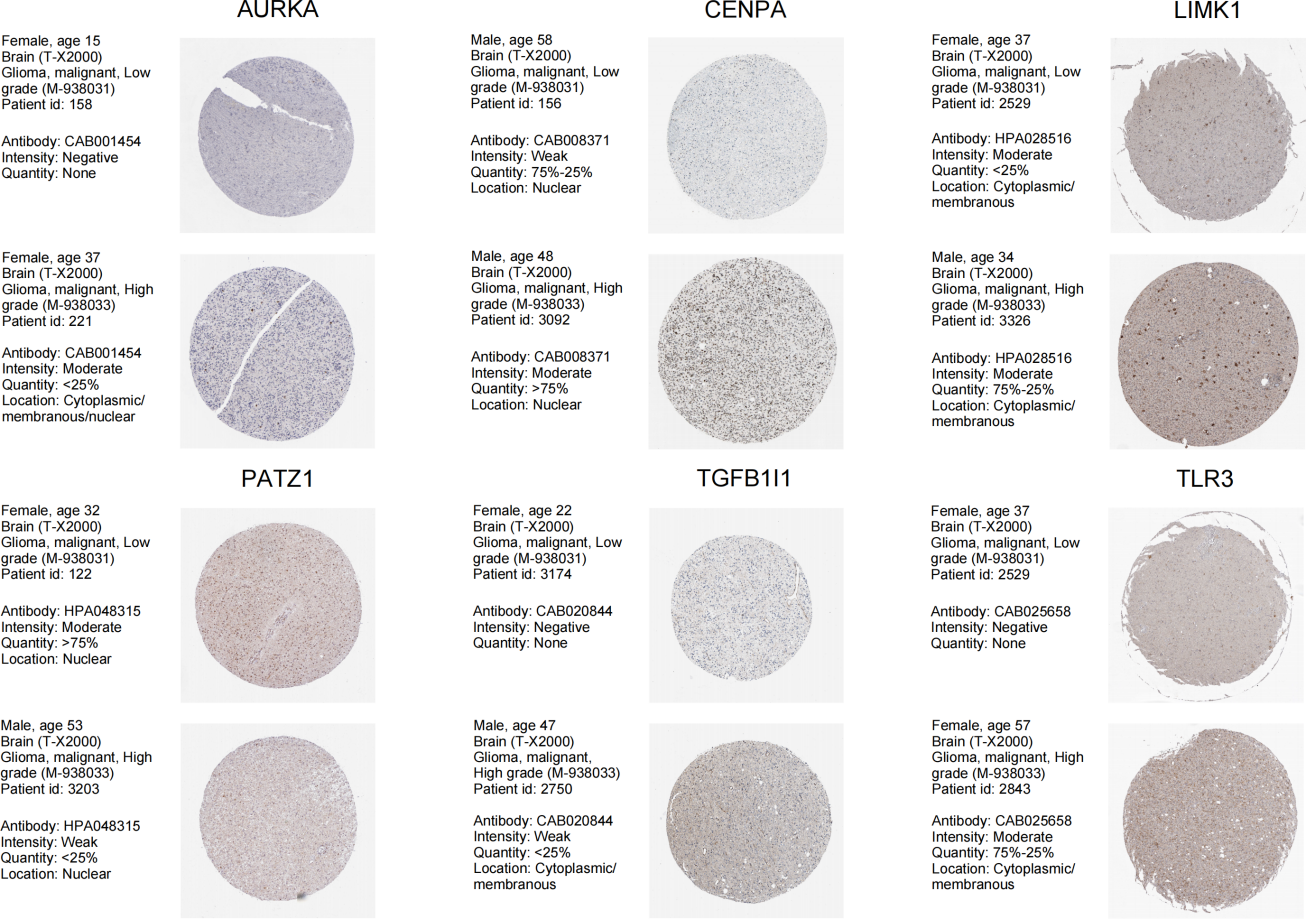
Immunohistochemical analyses from HPA database of the 6 SnGs in LGG and HGG, including AURKA, CENPA, LIMK1, PATZ1, TGFB1I1, and TLR3.

Univariate Cox analyses were used to identify the prognostic significance of the 65-differential expressed SnGs in LGGs. A total of 38 genes have been identified to be correlated to the OS. The expression level of these genes has been confirmed by immunohistochemical analyses in Human Protein Atlas (http://www.proteinatlas.org/). Then the genes which were untested or had poor consistency with RNA expression data were excluded. There were 18 genes were subsequently included in LASSO regression analysis, and 6 SnGs were eventually selected to establish the SnG signature, including AURKA, CENPA, LIMK1, PATZ1, TGFB1I1, TLR3 (**Figures 2A, B**). The regression coefficients for each gene have been shown (**Table 1**). Then we assessed the possible correlations between these 6 SnGs. The results showed a high correlation between AURKA and CENPA (r=0.854, P<0.001), and the other ratios were weakly to moderately correlated (**Figure 2C**). With the increase of grade, the expressions of AURKA, CENPA, LIMK1, TGFB1I1, and TLR3 were significantly up-regulated, while the expression of PATZ1 was down-regulated (**Figures 2D, E**). Kaplan-Meier analyses showed that AURKA, CENPA, LIMK1, TGFB1I1, and TLR3 were negatively associated with the outcome, whereas PATZ1 was positively associated with the outcome (**Figure 2F**). The immunohistochemical results of these 6 genes have been shown (**Figure 3**). The expressions levels of AURKA, CENPA, LIMK1, TGFB1I1, and TLR3 were lower in LGG than HGG samples, whereas the expression level of PATZ1 was higher in LGG. We obtained the corresponding risk scores of each patient according to the formula mentioned and separated the patients into two different risk groups.

**Table 1:** Senescence-related genes, the relationship with OS, and the coefficients in LASSO regression model.

Gene	Description	Hazard ratio (95% CI)	P value	Coefficients
AURKA	Aurora Kinase A	1.748 (1.521-2.010)	<0.001*	0.129695
CENPA	Centromere Protein A	1.582 (1.412-1.772)	<0.001*	0.301414
LIMK1	LIM Domain Kinase 1	1.907 (1.481-2.456)	<0.001*	0.057793
PATZ1	POZ/BTB And AT Hook Containing Zinc Finger 1	0.538 (0.405-0.716)	<0.001*	-0.753527
TGFB1I1	Transforming Growth Factor Beta 1 Induced Transcript 1	1.796 (1.469-2.195)	<0.001*	0.154034
TLR3	Toll Like Receptor 3	1.906 (1.554-2.336)	<0.001*	0.362497

*P<0.05, significant difference.

**Figure 4:**
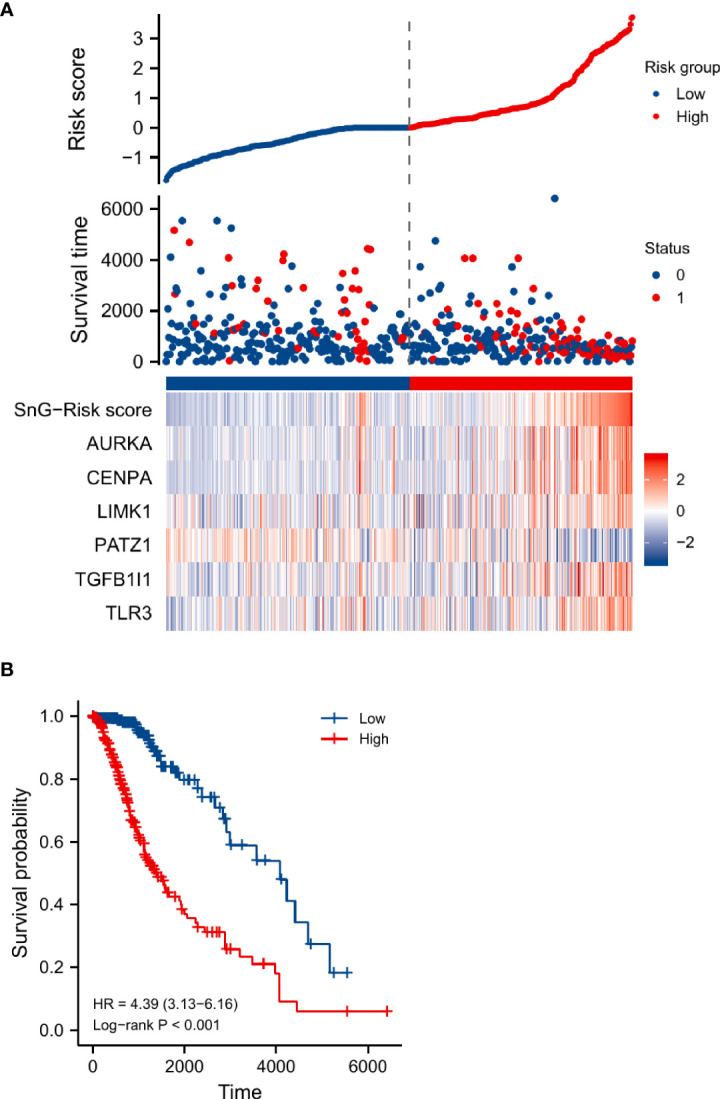
The survival distribution, risk score, and heat-map of gene-expression levels of the SnG-signature. **(A)** The survival distribution, risk score, and expression levels of the SnG-signature. **(B)** Kaplan-Meier analysis for estimation of the OS with different risk levels in LGGs.

We analyzed the survival distribution and expression profiles of the 6 SnGs between the two risk groups (**Figure 4A**). The result of Kaplan-Meier analysis showed that the high-risk score indicated a worse prognosis in LGGs (P<0.001, **Figure 4B**). The result of Cox regression showed that the SnG-risk score was an independent indicator for LGG survival (HR=2.763, 95%CI=1.660-4.599, P<0.001, **Table 2**).

**Table 2:** Univariate and multivariate Cox regression analyses in LGGs.

Characteristics	Univariate analysis		Multivariate analysis	
	Hazard ratio (95% CI)	P value	Hazard ratio (95% CI)	P value
Age				
<40	Reference		Reference	
≥40	2.906 (2.008-4.205)	<0.001*	3.599 (2.306-5.619)	<0.001*
Gender				
Female	Reference			
Male	1.124 (0.800-1.580)	0.499		
WHO grade				
II	Reference		Reference	
III	3.059 (2.046-4.573)	<0.001*	1.737 (1.114-2.708)	0.015*
IDH status				
WT	Reference		Reference	
Mutant	0.186 (0.130-0.265)	<0.001*	0.434 (0.271-0.693)	<0.001*
1p/19q codeletion				
Non-codel	Reference		Reference	
Codel	0.401 (0.256-0.629)	<0.001*	0.623 (0.367-1.057)	0.079
Risk score				
Low	Reference		Reference	
High	4.473 (2.978-6.718)	<0.001*	2.763 (1.660-4.599)	<0.001*

WT wild type; Mut mutant; Codel codeletion; Non-codel non-codeletion.

*P<0.05, significant difference.

### Risk score in different subgroups

**Figure 5:**
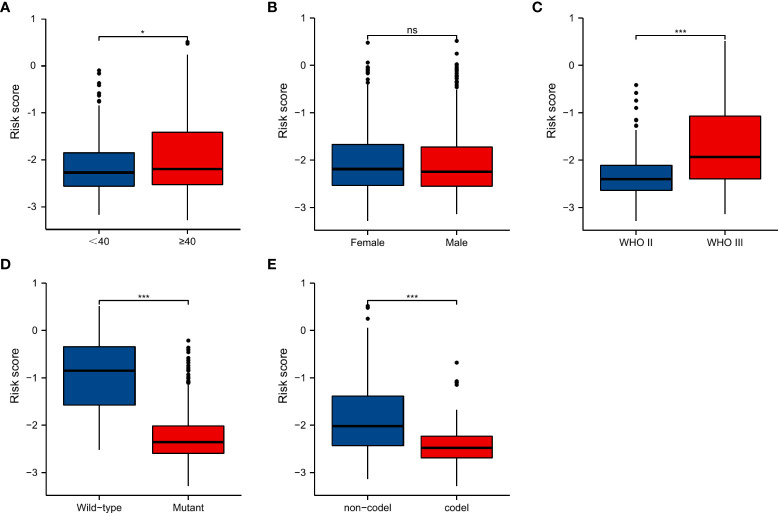
SnG-risk score distributions in LGGs stratified by different clinicopathologic features, including age, gender, WHO grade, IDH status, and 1p/19q co-deletion status. *P<0.05; ***P<0.001. **(A)** Age, **(B)** Gender, **(C)** WHO grade, **(D)** IDH status, **(E)** 1p/19q co-deletion status. ns, not significant.

We evaluated the SnG-risk score with different clinical characteristics (**Figures 5A–E**). The results showed that it significantly enhanced in groups of elder (P<0.05), WHO grade III (P<0.001), IDH wild-type (P<0.001), and 1p/19q non-codeletion (P<0.001).

### Nomogram development and validation

**Figure 6:**
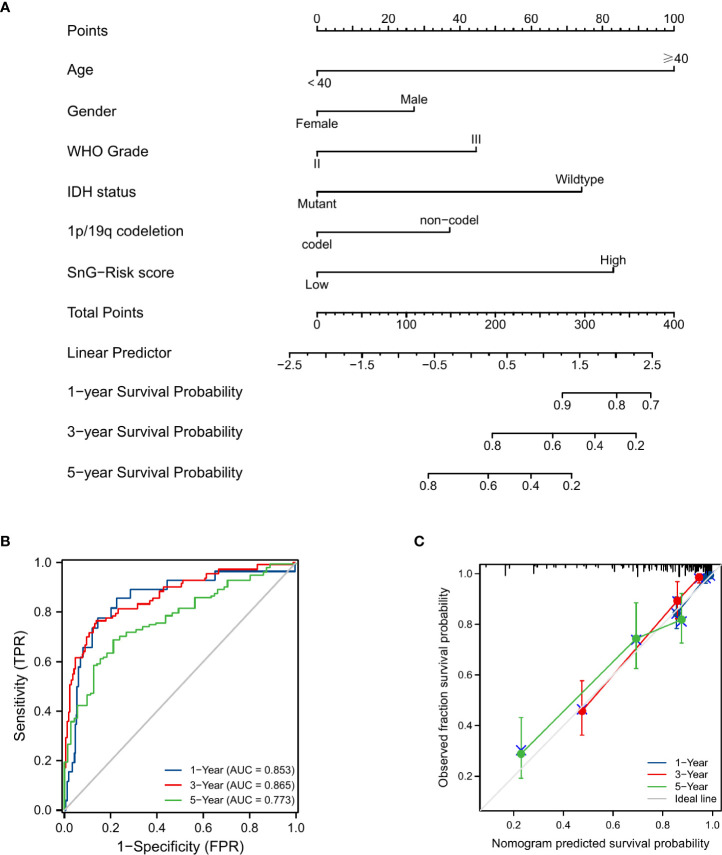
Prognosis prediction model for LGG patients. **(A)** Nomogram for 1-, 3-, and 5-year OS rates. **(B)** Time-dependent ROC curves of the nomogram. **(C)** Calibration plots of the nomogram.

We involved the same clinical characteristics of the Cox regression into the nomogram (**Figure 6A**). The concordance index of nomogram was 0.827 (95%CI=0.806-0.848). The AUCs of time-dependent ROC curves were 0.853, 0.865, and 0.773 for 1-, 3-, and 5-year OS rates, respectively (**Figure 6B**). And the predicted probability of calibration plots agreed with the observed results (**Figure 6C**).

### GSEA analyses

**Figure 7:**
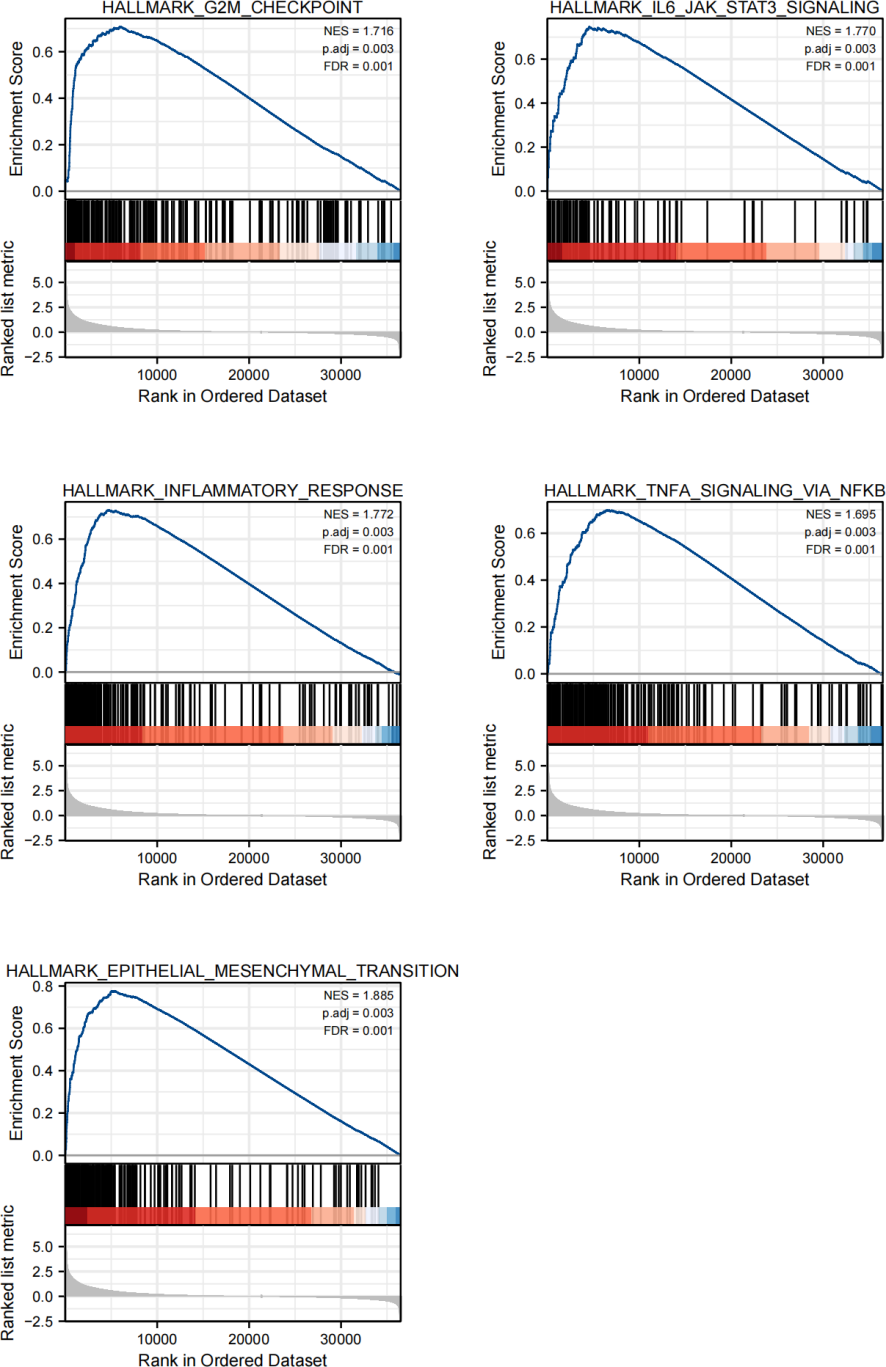
GSEA of the SnG-signature. G2M checkpoint, IL6/JAK/STAT3 signaling, Inflammatory response, TNFα signaling *via* NFκB, Epithelial mesenchymal transition.

We performed GSEA analyses to further identify the biological function involved in LGGs with different SnG-related risk score levels. The results revealed that G2M checkpoint, IL6/JAK/STAT3 signaling, inflammatory response, TNFα signaling *via* NFκB, and epithelial mesenchymal transition (EMT), were enriched in the high-risk group (**Figure 7**).

### Immunological analyses

**Figure 8:**
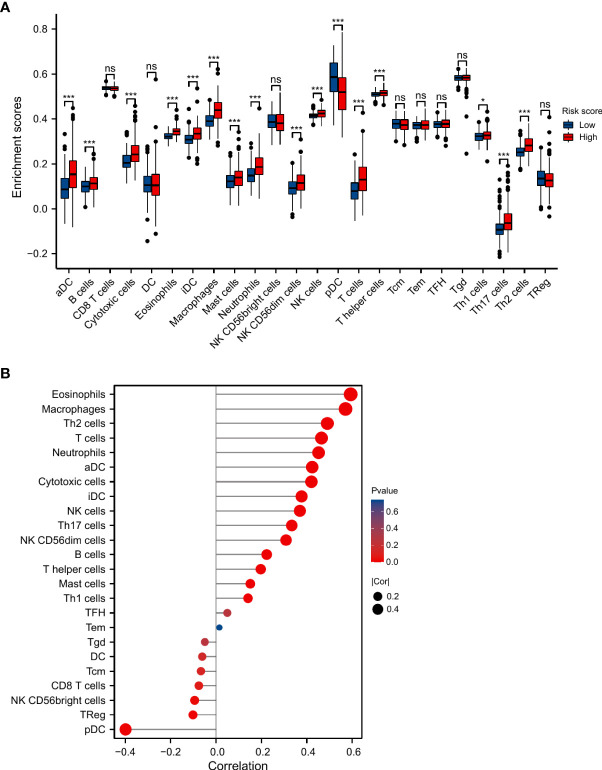
Association between SnG-risk score and immune infiltration in LGGs. **(A)** Infiltrating levels of different types of immune cells in low and high risk groups. **(B)** Correlation between SnG-risk score and immune cells. *P<0.05; ***P<0.001; ns, not significant.

We compared the infiltration level of immune cells between the two risk groups (**Figure 8A**). It showed that the levels of aDCs (activated dendritic cells), B cells, cytotoxic cells, eosinophils, iDCs (immature DCs), macrophages, neutrophils, NK CD56dim cells, NK cells, T cells, T helper cells, Th1, Th17 cells, and Th2 cells were significantly higher in the high-risk group, while pDCs (plasmacytoid DCs) were significantly decreased. And there were positive correlations between risk score and infiltration levels of eosinophils, macrophages, Th2 cells, T cells, neutrophils, aDCs, cytotoxic cells, iDCs, NK cells, Th17 cells, NK CD56dim cells, B cells, T helper cells, mast cells, Th1 cells. The negative correlations were found in NK CD56bright cells, TReg, and pDCs (**Figure 8B**).

**Figure 9:**
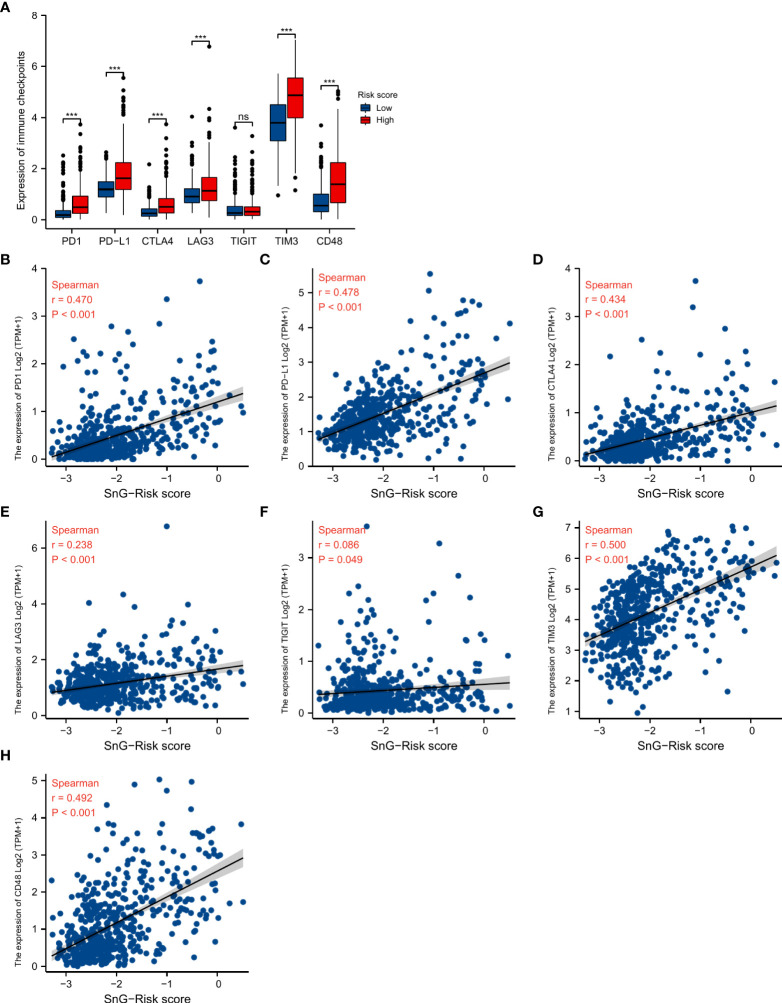
Association between SnG-risk score and immune checkpoint expression. **(A)** The expression levels of immune checkpoints in low and high risk levels. **(B–H)** Correlation between SnG-risk score and expression levels of immune checkpoints. *P<0.05; ***P<0.001; ns, not significant.

We analyzed the expression of immune checkpoints between the two groups, and evaluated the association between SnG-risk score and immune checkpoints (**Figures 9A–H**). The result showed that the expression levels of immune checkpoints were significantly higher in high-risk group. And there were significantly positive correlations between the expression of PD1, PD-L1, CTLA4, LAG-3, TIM3, CD48 and risk score.

### Validation on the prognostic value of the SnG-signature

**Figure 10:**
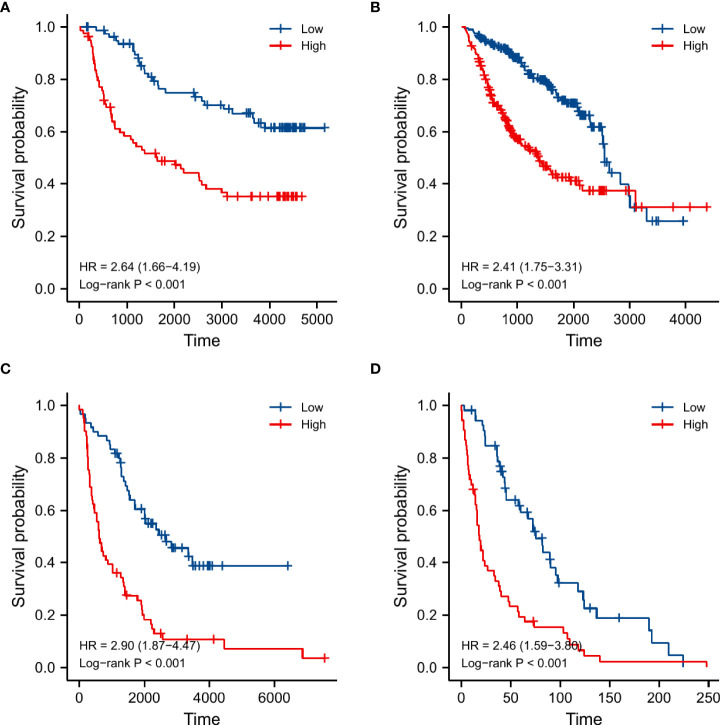
Kaplan-Meier curves and log-rank tests used for survival analyses between low and high risk groups in different validation datasets. **(A)** CGGA microarray dataset, **(B)** CGGA sequencing dataset, **(C)** REMBRANDT cohort, **(D)** GSE16011 dataset.

We validated the prognostic role of the SnG-risk system in three databases. The Cox regression analyses by CGGA microarray dataset (**Table 3**) and CGGA sequencing dataset (**Table 4**) have verified that the SnG-risk score played an independent role in LGG survival prognosis. And the Kaplan-Meier analyses in CGGA microarray dataset (**Figure 10A**), CGGA sequencing dataset (**Figure 10B**), REMBRANDT cohort (**Figure 10C**), and GSE16011 dataset (**Figure 10D**) suggested that the high-risk score indicated a poor outcome (P<0.001 for all).

**Table 3:** Univariate and multivariate Cox regression analyses in CGGA microarray dataset.

Characteristics	Univariate analysis		Multivariate analysis	
	Hazard ratio (95% CI)	P value	Hazard ratio (95% CI)	P value
Age				
<40	Reference			
≥40	1.452 (0.920-2.292)	0.109		
Gender				
Female	Reference			
Male	1.113 (0.702-1.764)	0.649		
WHO grade				
II	Reference			
III	3.091 (1.952-4.896)	<0.001*	0.961 (0.338-2.731)	0.941
IDH status				
WT	Reference			
Mutant	0.615 (0.383-0.989)	0.045*	1.228 (0.480-3.144)	0.668
1p/19q codeletion				
Non-codel	Reference			
Codel	0.243 (0.083-0.715)	0.010*	0.230 (0.074-0.714)	0.011*
Risk score				
Low	Reference			
High	2.445 (1.515-3.947)	<0.001*	5.289 (1.781-15.708)	0.003*

WT wild type; Mut mutant; Codel codeletion; Non-codel non-codeletion.

*P<0.05, significant difference.

**Table 4:** Univariate and multivariate Cox regression analyses in CGGA sequencing dataset.

Characteristics	Univariate analysis		Multivariate analysis	
	Hazard ratio (95% CI)	P value	Hazard ratio (95% CI)	P value
Age				
<40	Reference			
≥40	1.258 (0.915-1.728)	0.157		
Gender				
Female	Reference			
Male	1.007 (0.734-1.381)	0.965		
WHO grade				
II	Reference			
III	2.544 (1.780-3.635)	<0.001*	2.819 (1.896-4.193)	<0.001*
IDH status				
WT	Reference			
Mutant	0.459 (0.325-0.647)	<0.001*	0.741 (0.495-1.109)	0.145
1p/19q codeletion				
Non-codel	Reference			
Codel	0.353 (0.231-0.541)	<0.001*	0.543 (0.339-0.870)	0.011*
Risk score				
Low	Reference			
High	2.456 (1.773-3.403)	<0.001*	2.078 (1.401-3.082)	<0.001*

WT wild type; Mut mutant; Codel codeletion; Non-codel non-codeletion.

*P<0.05, significant difference.

## Discussion

Gliomas were common intracranial malignant neoplasms (32). Due to the heterogeneity in natural processes and molecular characteristics, the clinical outcome of LGGs varied widely (33, 34). Even with the current treatment strategy, the malignant progression and local recurrence of LGGs seemed to be inevitable. Developing a new prognostic model and exploring promising therapeutic biomarkers for LGG patients have been necessary. Cellular senescence has been considered a failsafe program for the organism triggered by severe interior or exterior damage (35). SASP-related immune response eliminated the further expansion of deleterious cells and suppressed the tumorigenesis, imposing an antitumor effect (36, 37). Conversely, the release of SASP factors led to the establishment of an immunosuppressive and chronic inflammatory microenvironment, which promoted tumor growth and chemotherapy resistance (38, 39). A recent study identified three senescence-related subtypes (C1, C2, and C3) in LGGs with the TCGA database. Then the researchers constructed the risk model with the 6 differentially expressed genes between the different subgroups, including TMSB4X, CDK6, FOXM1, IGFBP5, ITGB4, and IGFBP3. The results indicated the prognostic role of the risk model in LGGs (40). However, the role of cellular senescence needed to be further investigated, and its predictive value in clinical prognosis of LGG needed to be verified by large samples from multiple databases. Thus, we selected 6 differentially expressed SnGs (AURKA, CENPA, LIMK1, PATZ1, TGFB1I1, TLR3) to establish the signature. The expression of the 6 SnGs included in our study has been confirmed by IHC results. The predictive role of the SnG-signature has been validated with a total of 1416 samples from five different datasets (TCGA, CGGA microarray, CGGA sequencing, REMBRANDT, and GSE16011).

We first selected 279 experimentally validated SnGs from the CellAge database, of which 65 were considered to be differentially expressed. We explored the gene function with enrichment analyses. The GO analyses suggested that the genes mainly participated in cell cycle and transcription regulation. And the KEGG analysis showed the genes were correlated with cellular senescence and glioma.

By further univariate Cox analysis and LASSO regression, 6 SnGs were identified as potential prognostic markers and enrolled in the risk system. And we found that the expression levels of five genes (AURKA, CENPA, LIMK1, TGFB1I1, TLR3) had a negative correlation with the prognosis, whereas PATZ1 had a positive correlation. AURKA was a serine/threonine kinase driving cell-cycle progression by promoting cyclin B1, Wnt, myc, and other pro-proliferative pathways (40, 41). The amplification and overexpression of AURKA were commonly observed in human malignancies, which led to mitotic assembly checkpoint override and induced resistance in chemotherapy (42, 43). CENPA was a centromere-specific histone-H3-like variant gene. It played an important role in chromosome segregation regulation during cell division (44, 45). CENPA was thought to be involved in the nucleosomal packaging of centromeric DNA (46). Experiments have verified that CENPA overexpression contributed to the proliferation and metastasis of clear cell renal cell carcinoma by accelerating the cell cycle and activating the Wnt/β-catenin signaling pathway (47). LIMK1 was a member of serine/threonine kinase family and was highly expressed in various tumors. The enhancement of LIMK1 expression promoted cervical cancer progression (48). The down-regulation of LIMK1 could inhibit the growth of lung cancer and GBM (49, 50). PATZ1 encoded a transcription factor that belonged to the BTB/POZ group of transcriptional regulators (51). PATZ1 could bind p53-dependent gene promoters, enhancing p53-dependent transcription and apoptosis (52). PATZ1 colocalized intracellularly with PUMA, inhibiting cell proliferation and inducing apoptosis through PUMA in GBM (53). TGFB1I1 was a TGFβ-responsive gene which was also known as Hic-5, and it was involved in the cellular response to vascular injury (54). TGFB1I1 was found to be associated with TGFβ stimulated EMT process in the malignant progression of astrocytomas (55). Moreover, the high expression of TGFB1I1 suggested the sensitivity of advanced colorectal cancer to chemotherapy (56). TLR3 was abundantly expressed by the central nervous system cells, and it played a crucial role in innate immune and inflammation (57, 58). TLR3 might contribute to the protection of cisplatin-induced DNA damage response leading to head and neck cancer development and cisplatin resistance (59).

Based on the expression of 6 SnGs, the risk scores of each LGG sample were calculated. The Cox regression indicated the prognostic role of SnG-risk score for LGG patients. The Kaplan-Meier analysis revealed that the high SnG-risk score level correlated to a worse outcome in LGGs. And we found the risk score enhanced in elder, WHO grade III, IDH wild-type, and 1p/19q non-codeletion groups (P<0.05 for all), which was consistent with the prognostic significance of these clinical factors. Then we integrated the same items of Cox regression into the prognostic nomogram model for predicting the OS rates. Furthermore, we performed GSEA analyses, and the results showed enriched phenotypes of G2M checkpoint, immune responses, and EMT in high-risk groups. A previous study has found that the induction of arrest in G2M cell cycle phase could lead to significant attenuation in cell migratory and invasion indices of LGGs (60). The activation of IL6/JAK/STAT3 pathway between tumor-initiating cells and macrophages has been shown to lead to poor outcomes in glioma patients (61). Another study has found that KDM6B could affect the EMT process in glioma cells by regulating a senescence-related gene SNAI1 (62).

Immune infiltration in tumor microenvironment was considered to be associated with tumorigenesis and progression. So we analyzed the correlation between SnG-signature and immune cells in LGG samples. And we observed that eosinophils (r=0.593, P<0.001), macrophages (r=0.570, P<0.001) had the highest correlation with SnG-risk score. Eosinophils produced matrix metalloproteinases, amphiregulin, TGF-α, or other growth factors in response to tumor-derived GM-CSF. The increase in epidermal growth factor ligands expression induced the GM-CSF production of glioma cells, developing a paracrine loop, which promoted the process of glioma (63). The increased infiltration of macrophages might indicate that the phenotypic transformation of tumor-associated macrophages drove the immune microenvironment to an immunosuppressive state, which reduced the inflammatory immune response (64). In addition, the high infiltration of Th2 cells (r=0.490, P<0.001), T cells (r=0.464, P<0.001), and innate immune cells, neutrophils (r=0.451, P<0.001), also contributed to the excessive immune responses and dysregulated immune microenvironment, which might lead to the shorter OS in the high-risk group. Moreover, we observed significantly positive correlations between immune checkpoint expression and the SnG-risk score. The above results suggested that SnGs might have the potential to predict the efficacy of immunotherapy in LGG patients individually. The predictive value of our model has been verified in several datasets, including CGGA microarray dataset, CGGA sequencing dataset, REMBRANDT dataset, and GSE16011 dataset. However, our study still had some limitations. The potential signaling pathway of these genes should be explored by experiments in the following studies. And the predictive value of the signature needs further validation in large-scale studies.

## Conclusion

We established a prognostic model for LGG patients based on senescence-related genes and validated its predictive value in three external databases. The high SnG-risk score indicated a poor prognosis in LGGs. Our study has suggested that the selected genes were promising to become potential therapeutic targets for LGG treatment. And we will further study the regulatory mechanism and signaling pathway in the future.

## Data availability statement

The original contributions presented in the study are included in the article/supplementary material. Further inquiries can be directed to the corresponding authors.

## Ethics statement

We obtained all the RNA-seq data and the matching clinical features from public databases. And informed consents were obtained from all participants according to the publication guidelines of these databases. The Ethics Committee in Beijing Tiantan Hospital approved this study.

## Author contributions

JL illustrated all the results and drafted the manuscript. WW performed the GO, KEGG, GSEA, and immune-related analyses. DL downloaded the data from the public databases. CT performed the statistical analyses. JW performed the revision of the manuscript. JZ conceived this research. All authors have revised and approved the final version of the manuscript.

## Funding

National Natural Science Foundation of China (82102757) supported this study.

## Conflict of interest

The authors declare that the research was conducted in the absence of any commercial or financial relationships that could be construed as a potential conflict of interest.

## Publisher’s note

All claims expressed in this article are solely those of the authors and do not necessarily represent those of their affiliated organizations, or those of the publisher, the editors and the reviewers. Any product that may be evaluated in this article, or claim that may be made by its manufacturer, is not guaranteed or endorsed by the publisher.
